# MiRNAs in β-Cell Development, Identity, and Disease

**DOI:** 10.3389/fgene.2016.00226

**Published:** 2017-01-11

**Authors:** Aida Martinez-Sanchez, Guy A. Rutter, Mathieu Latreille

**Affiliations:** ^1^Section of Cell Biology and Functional Genomics, Division of Diabetes, Endocrinology and Metabolism, Department of Medicine, Imperial College LondonLondon, UK; ^2^Cellular Identity and Metabolism Group, MRC London Institute of Medical SciencesLondon, UK; ^3^Institute of Clinical Sciences, Faculty of Medicine, Imperial College LondonLondon, UK

**Keywords:** microRNAs, gene expression, insulin, cellular identity, pancreatic β-cells, diabetes

## Abstract

Pancreatic β-cells regulate glucose metabolism by secreting insulin, which in turn stimulates the utilization or storage of the sugar by peripheral tissues. Insulin insufficiency and a prolonged period of insulin resistance are usually the core components of type 2 diabetes (T2D). Although, decreased insulin levels in T2D have long been attributed to a decrease in β-cell function and/or mass, this model has recently been refined with the recognition that a loss of β-cell “identity” and dedifferentiation also contribute to the decline in insulin production. MicroRNAs (miRNAs) are key regulatory molecules that display tissue-specific expression patterns and maintain the differentiated state of somatic cells. During the past few years, great strides have been made in understanding how miRNA circuits impact β-cell identity. Here, we review current knowledge on the role of miRNAs in regulating the acquisition of the β-cell fate during development and in maintaining mature β-cell identity and function during stress situations such as obesity, pregnancy, aging, or diabetes. We also discuss how miRNA function could be harnessed to improve our ability to generate β-cells for replacement therapy for T2D.

## Background

Pancreatic β-cells are responsible for insulin release and thus are essential for normal blood glucose homeostasis. Thanks to an unusual metabolic configuration (Rutter, 2001, 2004; Rutter et al., 2015), β-cells act as nutrient sensors by coupling oxidative glucose metabolism to insulin secretion, delivering an appropriate quantity of the hormone into the bloodstream at any given time. When metabolized by the β-cell, glucose promotes an increase in intracellular ATP/ADP ratio that induces the closure of ATP-sensitive K^+^ channels (K_ATP_) (Ashcroft and Rorsman, 2013) which in turn leads to the depolarization of the plasma membrane and the opening of voltage-gated Ca^2+^ channels. The resultant increase in intracellular free Ca^2+^ triggers both the exocytotic release of insulin from secretory granules and reinforces the response by stimulating further ATP production by mitochondria (Rutter et al., 2015). Other K_ATP_-independent mechanisms (Henquin, 2009), which are still incompletely understood but may include the inhibition of AMP-activated kinase (AMPK) and the action of fatty acid derivatives, potentiate the above signaling mechanism (da Silva Xavier et al., 2003; Prentki et al., 2013).

During the development of T2D, peripheral insulin resistance leads to hyperglycemia and initially triggers a compensatory response from the β-cells to release more insulin (Nolan et al., 2006). Frank diabetes presents when β-cells are incapable of compensating for insulin resistance, and this is associated with some loss of β-cell mass (Butler et al., 2003; Rahier et al., 2008), whose extent is debated (25–50%), compounded by impaired glucose-stimulated insulin secretion from the remaining β-cells (Meier and Bonadonna, 2013). Importantly, the first phase of insulin secretion is usually lost when fasting blood glucose levels are only slightly raised (∼6 mmol/L), suggesting that β-cell dysfunction is an early event in disease progression (Kahn et al., 2009).

Loss of β-cell identity (i.e., de-differentiation or trans-differentiation) has recently been suggested to be an important contributor to both the apparent loss of β-cell mass which may be the result of a failure to detect remaining β-cells in which insulin levels have fallen below threshold levels (Marselli et al., 2014) and to impaired function (Del Guerra et al., 2005). The latter phenomenon was first described in hyperglycemic and Zucker Diabetic Fatty rats (Tokuyama et al., 1995; Jonas et al., 1999), and is characterized by increased expression of normally repressed genes, such as hexokinase (*HKI-III*), and decreased levels of genes important for β-cell secretory function (e.g., *Glut2*/*Slc2a2*) as well as key transcription factors such as *Pdx1*. Moreover, in the mouse, genetic ablation of *FoxO1* caused β-cells to de-differentiate into progenitor-like cells and even α-cell-like cells following physiologic stress associated with insulin resistance (multiple pregnancies or aging) (Talchai et al., 2012). Likewise, *Nkx6.1* and *Pdx1*-deficient β-cells acquired the molecular characteristics of δ-cells and α-cells, respectively (Schaffer et al., 2013; Taylor et al., 2013; Gao et al., 2014). In certain cases, dedifferentiated, or so-called “empty” (i.e., depleted in dense core insulin-containing granules) β-cells, express markers solely found in endocrine progenitor cells (Talchai et al., 2012; Puri et al., 2013; Wang et al., 2014) presumably reflecting plasticity to allow reprogramming toward an alternate endocrine identity (Talchai et al., 2012; Taylor et al., 2013; White et al., 2013). Most recently, Spijker et al. (2015) presented evidence that loss of β-cell identity occurs in T2D in humans and primates, although its contribution to the development of diabetes remains contested (Butler and Dhawan, 2015; Butler et al., 2016; Md Moin et al., 2016; Wang Y.J. et al., 2016) with little evidence from studies of the islet transcriptome from subjects with T2D of increases in progenitor markers (Fadista et al., 2014).

β-cell identity is normally maintained at multiple levels, including through the action of transcriptional activators and/or repressors, epigenetic mechanisms (such as DNA methylation or histone modifications) and non-coding RNAs. De-regulation of any or all of these in the β-cell might thus be associated with the development of diabetes (McKinnon and Docherty, 2001; Avrahami and Kaestner, 2012; Gilbert and Liu, 2012; Pullen and Rutter, 2014; Dayeh and Ling, 2015).

The majority of the genome (∼98%) is transcribed to produce transcripts lacking protein-coding potential, including microRNAs (miRNAs). MiRNAs are 21–22 nucleotide-long molecules that silence gene expression post-transcriptionally. More than 2,000 miRNAs have been identified so far in humans which regulate virtually every aspect of cell biology, including development, proliferation, differentiation or metabolism (Sun and Lai, 2013). It is therefore not surprising that disruption of miRNA function contributes to many human diseases, including cardiovascular disorders, cancer and neurological dysfunction (Small and Olson, 2011; Mendell and Olson, 2012; Emde and Hornstein, 2014).

Micro RNAs are transcribed as longer precursors (pri-miRNAs), generally by polymerase II (Lee et al., 2004). Thus, the expression of a large subset of mammalian miRNAs may be transcriptionally linked to the expression of other genes, allowing for coordinate regulation of miRNA and protein expression (Schanen and Li, 2011). Pri-miRNAs are first processed in the nucleus by DROSHA and, in mammals, DGCR8, into a ∼70 nt hairpin, known as pre-miRNA. The pre-miRNA is then exported to the cytoplasm where it is further processed by DICER into a small (∼20 nt) RNA duplex. In general, one of the strands (known as the guide) will be incorporated within an Argonaute (Ago) protein into a miRNA-induced silencing complex (miRISC) while the other one (passenger strand) is released and degraded (Finnegan and Pasquinelli, 2013).

Most metazoan miRNAs direct miRISC to the target mRNAs by interacting with sites of imperfect complementarity to induce their degradation and/or inhibit translation, resulting in repression of its expression (Pasquinelli, 2012). In general, the most important region for target recognition comprises the nucleotides 2–8 of the miRNA- known as the “seed” region- and binding sites located in the 3′ UTR of the cognate mRNAs are more common (Bartel, 2009). The capacity of miRNAs to target several transcripts simultaneously, while a given transcript can be concomitantly targeted by several different miRNAs, suggests an ability to build a complex regulatory network for fine-tuning gene expression (Gurtan and Sharp, 2013). Thus, miRNAs may contribute to maintain cell function in spite of internal or external perturbations and have recently emerged as reinforcers of developmental transitions and cellular identities (Ebert and Sharp, 2012). In the adult, miRNAs contribute to the maintenance of cell identity in a variety of cell types (Li and Jin, 2010; Xin et al., 2013) and to changes in cell fate in cancer (Berdasco and Esteller, 2010). MiRNAs might suppress neuronal genes during endocrine cell maturation (Kanji et al., 2013) and silence transcriptional repressors in adult β-cells (Melkman-Zehavi et al., 2011).

In this review, we discuss firstly the role miRNAs play in the acquisition and maintenance of β-cell identity. We then go on to describe how dysregulation of miRNA networks in response to metabolic stress or cellular insults might contribute to the loss of β-cell identity and to the development of T2D.

## MiRNAs and the Acquisition of β-Cell Identity

The past few years have provided a substantial amount of information on the morphological changes occurring during early pancreas formation in the mouse (Jorgensen et al., 2007) and humans (Pan and Brissova, 2014; Jennings et al., 2015). In mice, pancreas development is initiated by two evaginations of the foregut endoderm at embryonic day 8.5 (E8.5). These so-called pancreatic dorsal and ventral buds arise from thickening of the pancreatic epithelium that undergoes branching morphogenesis and eventually fuse to form a single organ later in embryogenesis. Pancreatic progenitor cells are defined by the expression of several transcriptional regulators including *Tcf2*, *HNF6*, *Foxa2*, *Hb9*, *Pdx1*, *Ptf1a*, *Nkx2.2*, *Nkx6.1*, *Sox4*, *Gata6*, *Gata4*, and *Sox9* (Maestro et al., 2003; Cano et al., 2014) which will differentiate into three different cell types composing the pancreas: endocrine, exocrine, and ductal cells. The differentiation of the pancreatic endocrine lineage including insulin-producing β-cells is triggered by the transient activation of neurogenin3 (*Ngn3*), a basic helix-loop-helix (*bHLH*) transcription factors enhancing the expression of lineage committed transcriptional regulators such as Rfx6 (Smith et al., 2010; Soyer et al., 2010). Although *Ngn3* expression is gradually lost by E15.5, its downstream transcriptional activators enable the terminal differentiation of pancreatic β-cells into mature insulin-producing cells.

Analysis of conditional *Dicer1* null mice has revealed the importance of miRNAs in the regulation of pancreatic endocrine cell differentiation. Deletion of *Dicer1* selectively in the developing pancreas (e8.5) using a Pdx1-Cre deleter strain produced a deficiency of β-cells attributed to a marked decreased in the number of Ngn3^+^ endocrine progenitor cells (Lynn et al., 2007). This result indicated an important role of miRNAs in the specification of progenitors into the endocrine lineage of the pancreas. In contrast, Kanji et al. (2013) showed that mice born with specific deletion of *Dicer1* in Ngn3^+^ progenitors are morphologically indistinguishable from controls and present no alteration in endocrine cell mass. However, a few weeks after birth the latter animals develop a striking decrease in endocrine cell mass, which is associated with decreased insulin secretion and the appearance of hyperglycemia. A further fascinating observation is the de-repression of several neuronal genes in neonatal Dicer1^Ngn3-cre^ islets including *Rest1*, tyrosine hydroxylase (*Th*), *Phox2a* and *Phox2b*. This indicates that *Dicer1* is dispensable for the specification of endocrine progenitors as hormone-producing cells but highlights a crucial role of miRNAs in maintaining β-cell identity by repressing a neuronal gene program (Kanji et al., 2013). Kalis et al. (2011) reported that conditional inactivation of Dicer1 in differentiated β-cells using Rip-Cre transgenic mice doesn’t affects β-cell mass in newborn mice. However, at 12-week of age, these mutant mice gradually developed hyperglycemia from 12 weeks, glucose intolerance and full-blown diabetes mellitus, which is attributed to impaired insulin secretion and loss of β-cell mass (Kalis et al., 2011; Mandelbaum et al., 2012).

**FIGURE 1:**
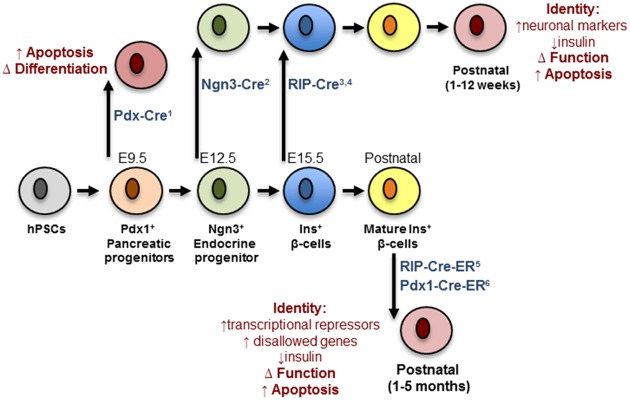
**Impact of Dicer depletion on β-cell maturation and maintenance.** Progenitors and mature β-cells are represented in different colors. The deleter strains are indicated in blue and contain references to the corresponding papers: (1) Lynn et al. (2007); (2) Kanji et al. (2013); (3) Mandelbaum et al. (2012); (4) Kanji et al. (2013); (5) Melkman-Zehavi et al. (2011); (6) Martinez-Sanchez et al. (2015). The black arrows mark the moment at which deletion occurs. Red cells represent defective cells and the biological pathways/functions affected are indicated in red. hPSC, human pluripotent stem cell.

Taken together, the above loss-of-function studies demonstrate a role for *Dicer1* and miRNAs in the early stages of pancreatic cell lineage differentiation (**Figure 1**). Nonetheless, they provide little information as to the role of specific miRNAs in the differentiation of β-cells. Initial small RNA cloning studies by Poy et al. (2004) revealed the existence of a diverse miRNA transcriptome in the MIN6 insulinoma cell line that included the highly expressed miR-375 (Pullen et al., 2011). Many other groups have subsequently confirmed high expression of miR-375 in adult mouse (Landgraf et al., 2007; Avnit-Sagi et al., 2009; Poy et al., 2009) and human (van de Bunt et al., 2013) islets as well as purified β-cells (Klein et al., 2013). Other profiling studies performed in the developing pancreas identified a set of miRNA whose expression was altered as the differentiation of pancreatic endocrine cells proceeds. In humans these include, amongst others, miR-7, -9, -15a/15b/16/195, -124a, -195, -218, -195, -375, -376a, -503, and -541 (Correa-Medina et al., 2009; Joglekar et al., 2009a; Sun and Lai, 2013). Conversely, e14.5 mouse pancreas shows high levels of let-7a, miR-136, -214, -375, -503, -541 (Lynn et al., 2007) whereas rat e20 pancreas hast high levels of miR-21, -23a, -29a, -125b, -376b, and -451 (Larsen et al., 2011).

Although, little genetic evidence exists demonstrating a role for the above specific miRNAs in pancreas genesis, they may regulate the acquisition of β-cell identity during early embryogenesis. In fact, miR-375, is also expressed in endodermal progenitor cells. Moreover, inhibition of miR-375 by morpholino oligonucleotides inhibits pancreatic islet development in *Xenopus laevis* (Kloosterman et al., 2007). The importance of miR-375 in regulating β-cell mass is also conserved in mice where global genetic inactivation of miR-375 results in decreased β-cell mass and diabetes (Poy et al., 2009; Latreille et al., 2015). MiR-375 expression levels also increased during pancreas development in humans (Joglekar et al., 2009a). Interestingly, the miR-375 promoter contains highly conserved binding sites for the transcriptional regulators hepatocyte nuclear factor 6 (*HNF6*) and Insulinoma-assoicated-1 (*INSM1)*, two critical components of the transcriptional cascade regulating Ngn3-dependent endocrine progenitor differentiation (Jacquemin et al., 2000; Avnit-Sagi et al., 2009, 2012; Zhang et al., 2009). PDX1, NGN3 and NEUROD1 binding sites are also found in enhancer regions required for full transcriptional activity of the miR-375 gene (Keller et al., 2007; Avnit-Sagi et al., 2009). Interestingly, several predicted mRNA targets of miR-375 such as *Gata6*, *Hnf1α*, and *Pax6* play important roles during the specification of pancreatic progenitors and terminal maturation of endocrine cells indicating that a miR-375 circuit directs appropriate β-cell development through temporal controls of β-cell transcription factor expression. Characterization of conditional mice with stage-specific inactivation of miR-375 in pancreatic and endocrine progenitors should help clarify the physiological role of miR-375 in β-cell differentiation.

**FIGURE 2:**
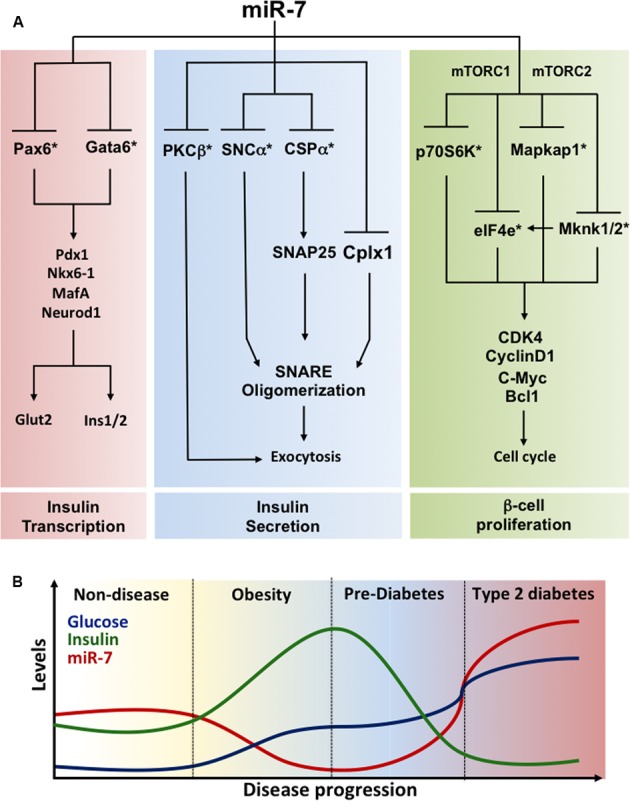
**Regulation of β-cell identity by miR-7** **(A)** miR-7 gene circuits prevailing in pancreatic β-cells. Functional analyses revealed that miR-7 controls three essential axes maintaining the identity of pancreatic β-cells: (1) Insulin gene transcription (left); (2) Insulin secretion (middle); and (3) β-cell proliferation (right). miR-7 represses Pax6 and Gata6 mRNAs, two transcription factors modulating insulin gene transcription. miR-7 also controls the expression of PKCβ, a Ser/Thr protein kinase activated following Ca^2+^ release, and SNCα and CSPα, two components of the exocytosis machinery modulating SNARE activity and the late step of insulin granule fusion with the plasma membrane. Finally, evidence indicate that miR-7 is a negative regulator of β-cell proliferation by repressing the expression of core components of the mTOR signaling pathways including p70S6K, eIF4e, Mapkap1, and Mknk1/2. By concomitantly repressing targets of these three axes, miR-7 couples rates in insulin transcription and secretion to β-cell proliferation. ^∗^Denotes a direct target of miR-7. **(B)** Regulation of miR-7 expression mediates the functional adaptation of β-cells to metabolic stress. Schematic illustrating the progressive changes in glucose (blue) and insulin (green) concentrations as well as in islet miR-7 expression (red) during the physio-pathological progression of type 2 diabetes. Fluctuations of these parameters are supported from data obtained with miR-7 mutants mice, mouse models of obesity and diabetes, as well as observations made in primary human islets and obese and diabetic patients. Data source: Esguerra et al. (2011), Kredo-Russo et al. (2012), Wang et al. (2013a), Latreille et al. (2014). See the main text for further details.

Kredo-Russo et al. (2012) also provided evidence for a role of miR-7 during pancreas development. Overexpression of miR-7 in pancreatic progenitors impaired differentiation of α- and β-cells and correlated with a repression of *Pax6* gene expression. These mutant mice displayed a similar phenotype to *Pax6* knockout animals. A comprehensive review of the function of miR-7 in regulating the identity of β-cells is presented below in the Case Study (see **Box 1** and **Figure 2**).

**MiR-7 as a regulator of β-cell identity.**

**MiR-7 gene family.** MiR-7 is amongst the most highly conserved miRNAs during evolution. In mice, three independent genomic loci encode miR-7: miR-7-al, miR-7-a2, and miR-7b (miR-7-1, miR-7-2, and miR-7b in humans). Unlike the intergenic miR-7a-2 and miR-7b genes, miR-7a-1 is found on chromosome 13 inside an intron linking exon 15 and 16 of the *HNRNPK* gene. In humans, all three mature miRNAs have identical nucleotide sequence whereas in mice and rats, miR-7b is distinct from the other two family members by a single substitution following the regulatory seed sequence mediating mRNA target selectivity. MiR-7 is a typical neuroendocrine miRNA expressed at high levels in pancreatic islets, pituitary, hypothalamus, and adrenals in mice and humans (Landgraf et al., 2007). Whereas little is known about the regulation of miR-7 gene transcription in these tissues, evidence for regulation of the processing of the primary transcript by RNA binding proteins has been demonstrated in various cell types (Lebedeva et al., 2011; Choudhury et al., 2013; Wang et al., 2013b). More recently, the ciRS-7 circular RNA, containing more than 70 highly conserved miR-7 binding sites and able to act as a sponge which titrates miR-7 activity, has been detected at high levels in brain (Hansen et al., 2013; Memczak et al., 2013). Interestingly, a recent report indicates that ciRS-7 is also expressed in pancreatic β-cells and regulates insulin gene transcription and secretion though change in miR-7 activity (Xu et al., 2015).

**Role of miR-7 in pancreas development.** Earlier studies (Wienholds et al., 2005) demonstrated that miR-7 is present in the developing pancreas in *Xenopus laevis*. In mammals, *in situ* hybridization revealed that miR-7 is predominantly expressed in insulin-, glucagon-, and somatostatin-producing cells of the developing and adult pancreas (Correa-Medina et al., 2009; Joglekar et al., 2009a; Kredo-Russo et al., 2012; Nieto et al., 2012). Furthermore, dynamic changes in miR-7 levels were found during pancreatic progenitor differentiation in mice (Nieto et al., 2012) and humans (Joglekar et al., 2009a). Similarly, miR-7 levels increase in human embryonic stem cells differentiated into insulin-producing cell *in vitro*, suggesting a role for the miRNA in the regulation of endocrine cell differentiation during development (Liao et al., 2013; Wei et al., 2013). Kredo-Russo et al. (2012) engineered conditional mice with a miR-7 gene targeted into the Rosa26 locus, which upon crossing with Pdx1-Cre mice allows for the overexpression of miR-7 in pancreatic progenitors. E15.5 mutant embryos displayed a decrease in proinsulin and proglucagon mRNAs. More investigation will be required to determine whether miR-7 affects endocrine lineage choice or the hormone contents in these cells. Interestingly, miR-7 expression is strikingly down-regulated in E14.5 Ngn3 knockout embryos (Kredo-Russo et al., 2012), suggesting that NGN3 drives endocrine cell differentiation through modulation of miR-7 expression.

**Function of miR-7 in adult mouse and humans β-cells.** MiR-7a-1 and miR-7a-2 conditional knockout mice were generated upon crossing floxed mice to RIP-Cre transgenic mice to achieve selective inactivation in the β-cell (Latreille et al., 2014). These investigations revealed improved glucose tolerance in miR-7a2 β-cell-specific knockout mice, which was associated with increased glucose-stimulated insulin secretion, findings recently confirmed by others (Xu et al., 2015). Electrophysiological measurements of capacitance changes revealed that miR-7 acts on the distal step of insulin granule fusion with the plasma membrane. Gene profiling analyses revealed that miR-7a represses several mRNAs encoding regulators of exocytosis and cytoskeleton reorganization including synuclein-a (Snca; Burre et al., 2010; Latreille et al., 2014). In contrast, mice overexpressing miR-7a-2 in β-cells developed pronounced diabetes from 4 weeks of age and this was associated with a decrease in circulating insulin levels due to a secretory deficit of β-cells (Latreille et al., 2014). Mutant mice also present with a striking down-regulation in the expression of insulin and of β-cell transcription factors including Pdxl, Nkx6.1, MafA, Neurodl, and Pax6. Given that Pax6 is directly repressed by miR-7 in β-cells (Kredo-Russo et al., 2012; Latreille et al., 2014) and Pax6 inactivation lowers insulin mRNA levels (Ahmad et al., 2015), the above suggests a pivotal role for the miR-7/Pax6 axis in triggering loss of β-cell identity in T2D. By regulating both the biosynthesis and secretion of insulin through Pax6 and Snca, respectively, miR-7 thus represents a node in a complex gene circuit regulating both the insulin transcription (i.e., β-cell identity) and secretion **(Figure 2A)**. Using dissociated adult primary β-cells, Wang et al. (2013a) demonstrated that anti-miR-7 oligonucleotide promotes β-cell replication *in vitro* by derepressing components of the mTOR signaling pathways **(Figure 2A)**. In contrast, loss- of function studies in mice failed to reveal a role for miR-7 in regulating the β-cell proliferation *in vivo* (Latreille et al., 2014). This could be explained by the methodology used or/and by the residual miR-7b levels found in characterized miR-7 knockout mice, which could mask an effect on β-cell proliferation. Further studies are warranted to clarify this discrepancy.

**Modulation of miR-7 expression by metabolic stress.** Given the physiological importance of miR-7 in regulating insulin exocytosis in β-cells, profiling of miR-7 expression was performed in mouse models of obesity and diabetes. miR-7a levels are decreased in islets from insulin resistant and obese mice (e.g., high fat feeding and ob/ob mice) that maintain euglycemia through a compensatory increase in insulin secretion. In contrast, islets from decompensating, hypoinsulinemic and diabetic db/db mice present increased expression of miR-7a, indicating that miR-7 gene induction accompanies pancreatic β-cell failure in diabetes. This further supports findings from Esguerra et al. (2011) showing increased levels of miR-7 in islets from non-obese GK diabetic rats. These observations were confirmed in healthy human islets transplanted under the kidney capsule of mice fed a high fat diet (Latreille et al., 2014). In the latter setting, miR-7a expression is down-regulated in transplants a few weeks after exposure to high fat diet, but gradually increases as metabolic stress impairs the ability of β-cells to secrete sufficient insulin to counteract peripheral insulin resistance (Latreille et al., 2014). Similarly, islets from obese pre-diabetic patients present with a ∼50% decrease in miR-7 levels (Latreille et al., 2014). On the other hand, quantification of miR-7 gene expression in islets from mildly diabetic patients (HbAlc 6.6 mmol/mol) surprisingly revealed lower miR-7 levels compared to controls, thus suggesting that the human response to metabolic stress in islets may differ from mice or alternatively that treatment of those patients may have allowed compensatory n-cell function to obesity and insulin resistance. Together, this reveals a dynamic remodeling of miR-7 expression during the natural progression of T2D, which contributes to the functional adaptation of β-cells to metabolic stress **(Figure 2B)**.

**Open questions**. These observations raise several questions on the physiological role of miR-7 in the pancreas. First, what is the precise biological function of miR-7 in healthy β-cells? One possibility is that it contributes toward buffering β-cell gene networks to provide functional robustness (Siciliano et al., 2013). Another possibility, although less attractive, is that miR-7 may mediate the functional adaptation of β-cells following a rapid elevation in glycaemia to prevent the over-secretion of insulin and hypoglycaemia. Due to the relatively high stability of miRNAs, it is unconceivable that prompt (sec–min.) fluctuation in insulin secretion could be explained by a change in miRNA expression. Second, is miR-7 induction the cause or simply yet another gene associated with β-cell failure and dedifferentiation in T2D? Third, what are the mechanisms underlying miR-7-induced dedifferentiation? Is this solely mediated by the single repression of Pax6 or caused by a combinatorial effect on multiple mRNA encoding for products maintaining the identity of β-cells? More investigation will be required to provide answers to these. This challenging endeavor will need to be fulfilled as it could provide innovating therapeutic opportunities leading to the development of anti-miR-7 agents to treating β-cell failure in T2D.

Fu et al. (2013) revealed that overexpression of miR-26a in mice increased the number of Ngn3^+^ endocrine progenitors and promoted their differentiation to into β-cells, findings that could be reproduced *in vitro* in sorted CD133+/Sox9+ progenitor-like cells. Several other groups reported that miRNAs inhibit the expression of multiple transcription factors driving β-cell differentiation. For example, miR-124a is abundant in e18 pancreas and represses *Foxa2* expression (Baroukh et al., 2007; Jing et al., 2014) suggesting that it may regulate the acquisition of the β-cell identity during development. MiR-124a also represses *Neurod1* in both MIN6 cells (Sebastiani et al., 2015) and neurons (Cheng et al., 2009; Liu et al., 2011). No genetic evidence for a role of this miRNA in the differentiation of pancreatic progenitor is available yet, but the relevance of miR-124a to the regulation of cellular identity is demonstrated by the suppression of non-neuronal genes by miR-124a in neuronal cells (Conaco et al., 2006). Interestingly, miR-124a expression is induced in islets from diabetic patients (Sebastiani et al., 2015) and single nucleotide polymorphisms (SNPs) in miR-124a gene have been identified in T2D islets (Ciccacci et al., 2013; Li Y. et al., 2015). Although expressed in low levels in adult islets, miR-124a is involved in the regulation of ATP-sensitive K^+^ channels, thus influencing glucose-stimulated Ca^2+^ dynamics (Baroukh et al., 2007).

Joglekar et al. (2007) also discovered that miR-15a and 15b were induced in regenerating, compared to developing, pancreas and found these to repress *Ngn3* via a post-transcriptional mechanism. By performing both gain- and loss-of-function experiments in mice, the above authors found that overexpression of miR15a/15b in pancreatic buds decreased *Ngn3* levels and reduced the number of α- and β-cells, whereas blocking miR-15 increased *Ngn3* levels after partial pancreatotectomy in mice led to higher levels of Nkx2-2 and NeuroD1, two of its downstream targets. *Rfx6*, another Ngn3-dependent gene in endocrine cells, is under tight regulation by two glucose-regulated miRNAs, miR-30d and let-7e (Liao et al., 2013). Others have shown that miR-19b represses insulin gene transcription following engagement of its mRNA target *NeuroD1* (Zhang et al., 2011). Nevertheless, a role for miR-19b, miR-30d, and let-7e during β-cell development remains to be demonstrated. Together, these findings highlight a complex interplay between miRNAs and transcription factors in β-cells. Future work in mice should help clarify how these interconnecting networks contribute to differentiation of β-cells in whole animals.

## Role of miRNAs in Maintaining Mature β-Cell Identity

Using genetic mouse models of DICER depletion, we and others have demonstrated that miRNAs are essential for the maintenance of mature β-cell identity, function, and survival (**Figure 1**). Thus, Melkman-Zehavi et al. (2011) were the first to examine the effects of disrupted DICER function in adult β-cells, using an inducible conditional RIP-CreER deleter strain. As early as 3 weeks after *Dicer* deletion, the null mice presented with marked hyperglycemia and glucose intolerance, mainly due to a severe reduction in β-cell insulin gene expression. Using a similar model and an inducible Pdx1-CreER deleter strain (Martinez-Sanchez et al., 2015) we proved that *Dicer* deletion eventually results in a strong decrease in β-cell mass. Most importantly, our studies demonstrated that the β-cell capacity to secrete insulin in response to a glucose challenge in miRNA-null islets was significantly impaired, a defect that preceded any loss in β-cell mass or insulin content. These defects, occurred concurrently with the up-regulation of β-cell “disallowed” genes, might contribute to loss of β-cell identity (see MiRNAs and Disallowed Genes). However, the expression of β-cell signature genes (e.g., *Pdx1*, *Nkx6.1*, *Nkx2.2*, *NeuroD1*, or *MafA*) was not altered in islets from either mouse model. Notably, Melkman-Zehavi et al. (2011) observed an up-regulation of the transcriptional repressors *Sox6* and *Bhlhe22*, associated with multipotency. Although the latter regulators are not ubiquitously expressed and do not, therefore, meet the criteria to be members of the “disallowed” gene family their expression is typically low in β-cells allowing efficient insulin transcription. Therefore, the up-regulation of these genes as a consequence of miRNA depletion might also contribute to the loss of β-cell identity.

As mentioned earlier, depletion of DICER in endocrine progenitors led to up-regulation of neuronal genes in early post-natal islets (Kanji et al., 2013). An increase in neuronal gene expression has also been observed in islets from mice with β-cell-specific deletion of LKB1 or AMPK (Kone et al., 2014) and contributes to the loss of β-cell identity, and possibly function, observed in these models. An up-regulation of neuronal genes following miRNA depletion in mature β-cells similarly occur and contribute to the failure of β-cell function in the latter setting. Nevertheless, this is an exciting possibility that remains to be explored.

Out of the 100s of miRNAs expressed in mature β-cells the function of only a small proportion has been deciphered so far. Specific miRNAs affect insulin production (Melkman-Zehavi et al., 2011; Zhang et al., 2011; Nieto et al., 2012; Setyowati Karolina et al., 2013; Xu et al., 2015), insulin exocytosis (Plaisance et al., 2006; Lovis et al., 2008a), growth (Tattikota et al., 2014), or apoptosis (Lovis et al., 2008b; Ruan et al., 2011). Additionally, to the best of our knowledge, the function of only four miRNAs (miR-375, miR-7a, miR-184, and the miR-200 family) has been addressed *in vivo* in the β-cell using mouse genetic studies (Poy et al., 2009; Latreille et al., 2014; Tattikota et al., 2014; Belgardt et al., 2015). This work has very recently been reviewed elsewhere (Filios and Shalev, 2015; Guay and Regazzi, 2016) and is not discussed here. Rather, we will focus on (1) the impact of miRNAs in β-cell identity and function through regulation of “disallowed” genes and (2) the role that miRNAs may play in loss of β-cell identity in situations of cellular stress, including diabetes, obesity, pregnancy, or aging.

## MiRNAs and Disallowed Genes

The normal secretory function of the β-cell requires the activation of several genes which are expressed in none or only a few other tissues. For example, the low affinity hexokinase, glucokinase (hexokinse type IV) is preferentially expressed in liver and β-cells and operates as a physiological glucose sensor thanks to its unique kinetic properties, different from those of hexokinases present in other tissues (Iynedjian, 2009). Conversely, several genes that serve a “housekeeping” role in most other cell types are poorly expressed or absent in β-cells. The two founder members of this group of “Disallowed” genes are the monocarboxylate transporter-1 (MCT-1/*Slc16a1*) and lactate dehydrogenase A (LDHA) (Sekine et al., 1994; Zhao et al., 2001). Previous studies have demonstrated that the low expression of those genes is likely, on one hand, to assure that pyruvate derived from glycolysis is preferentially directed toward mitochondrial oxidation – reinforcing the ability of glucose to stimulate insulin secretion- and, on the other hand, to avoid the stimulation of insulin secretion by the pyruvate generated by muscles during intense physical exercise (Pullen et al., 2012). Indeed, human mutations within the *SLC16A1* (MCT-1) promoter which increased its expression were found in families suffering from exercise-induced hyperinsulinemia (EIHI) (Otonkoski et al., 2007), revealing the importance of the absence of this transporter from β-cell for normal insulin secretion. Our group and others have subsequently identified ∼60 disallowed genes in the murine β-cell (Pullen et al., 2010; Thorrez et al., 2011), 11 of which were common in both of these studies. We have also recently demonstrated the importance of the silencing of another member of this family, the Acyl-CoA thioesterase 7 (Acot7) which β-cell specific overexpression lead to glucose intolerance and impaired insulin secretion in response to glucose (Martinez-Sanchez et al., 2016). These defects were associated with increased ATP consumption and decreased Ca^2+^ fluxes and insulin secretion in transgenic islets in response to glucose.

Interestingly, several disallowed genes have been found up-regulated in diabetic human islets (Marselli et al., 2010; Pullen and Rutter, 2013) and in islets from mouse models characterized by impaired β-cell identity such as those with β-cell specific deletion respectively of AMPK or *Rfx6* (Kone et al., 2014; Piccand et al., 2014).

**FIGURE 3:**
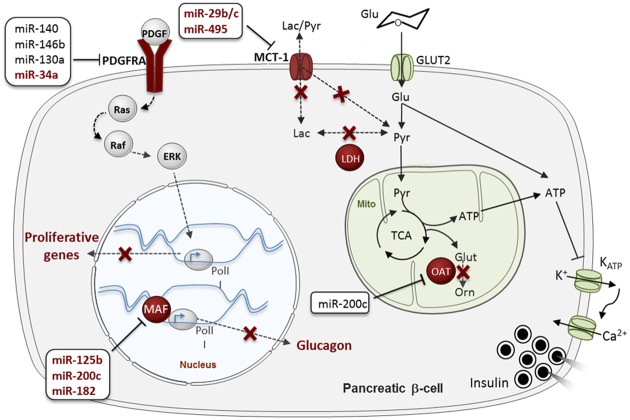
**MicroRNA (MiRNA)-mediated regulation of “Disallowed” genes in β-cells.** Disallowed genes are represented in red. The red crosses indicate pathways that are normally forbidden in β-cells thanks to the poor expression of the associated disallowed genes. MiRNAs regulating each disallowed gene are presented in boxes, in dark red (for validated miRNA-disallowed gene interactions) or black (for predicted targeting miRNAs), see main text. Glu, glucose; Pyr, pyruvate; Lac, lactate; Glut, glutamante; Orn, ornitine; PolII, RNA polymerase II; Mito, mitochondria. Data source: Pullen et al. (2011), Klein et al. (2013), Liang et al. (2015), Martinez-Sanchez et al. (2015), Tugay et al. (2016). See the main text for further details.

As occurs for β-cell-specific gene expression, disallowed gene repression is ensured at different levels. For example, the promoters of several disallowed genes, including those mentioned above, are enriched for repressor H3K27me3 histone marks in β-cells (van Arensbergen et al., 2010). As mentioned above, our group has recently demonstrated that miRNAs contribute to the *in vivo* silencing of 6 out of the 11 disallowed genes identified by both Pullen and Thorrez: *Slc16a1*, *Maf*, *Oat*, *Fcgrt*, *Igfbp4*, and *Pdgfra* (Martinez-Sanchez et al., 2015). Importantly, the up-regulation of the latter genes correlated with the loss of the secretory ability of the *Dicer*-depleted islets but preceded β-cell apoptosis, suggesting a role for gene disallowance in the former. Importantly, we demonstrated that five of those six disallowed genes (*Slc16a1*, *Maf*, *Oat*, *Fcgrt*, and *Pdgfra*) are directly targeted by miRNAs. As discussed below, the identity of those miRNAs has only been partially unraveled so far (**Figure 3**).

Pullen et al. (2011) have also demonstrated *in vitro* that miR-29b and miR-29c repress *Slc16a1*. Although, this gene is regulated by miR-124 in medulloblastomas (Li et al., 2009), miR-124 is not expressed at significant levels in mouse islets, at least in young adults, and is thus unlikely to impact *Slc16a1* expression in β-cells at this developmental stage (Pullen et al., 2011). More recently, Liang et al. (2015) showed that miR-495 targets *Slc16a1* in human embryonic stem cell (hESC) derived pancreatic endoderm that improved T2D symptoms upon transplantation in a murine model for T2D.

*Maf* is an activator of glucagon expression in α-cells that is repressed by miR-125b, miR-182 and miR-200c in MIN6 cells (Klein et al., 2013). Interestingly, the expression of *Maf* and its regulatory miRNAs is negatively correlated in β- and α-cells. A fascinating hypothesis is that alteration of those miRNAs, and others targeting this gene, contributes to β- to α-cell trans-differentiation.

*Pdgfra* is an important regulator of β-cell proliferation in mouse and humans as its repression contributes to limit the proliferative capacity of the adult β-cell (Chen et al., 2011). *Pdgfra* 3′ UTR is unusually long, characteristic of mRNAs subjected to tight post-transcriptional regulation and is predicted to be targeted by more than 50 conserved miRNAs^1^. Indeed, *Pdgfra* is an experimentally validated target for miR-34a in pulmonary artery smooth muscle and colon cancer cells and glioblastoma (Genovese et al., 2012; Li C. et al., 2015; Wang P. et al., 2016), miR-130a during mesodermal specification (Singh et al., 2015), miR-146b-5p in blood cells and hepatocellular carcinoma (Zhu et al., 2013; Zhai et al., 2014) and miR-140 during palatogenesis (Eberhart et al., 2008). Moreover, the Regazzi laboratory, in collaboration with ourselves, has very recently demonstrated that miR-34a targets *Pdgfra* in rat islet cells (Tugay et al., 2016). As discuss below, miR-34a expression increases with age in rat and human islets and might thus be involved in the age-related decline in β-cell proliferation through its target *Pdgfra* (Tugay et al., 2016). Interestingly, both miR-34a and miR-146 may impact β-cell apoptosis (Nesca et al., 2013) and miR-146b has also been implicated in the effect of cytokines on pancreatic β-cells (Roggli et al., 2010). Seyhan et al. (2016) found that both miR-34a and miR-146a are up-regulated in the circulation of patients with various forms of diabetes in comparison with healthy controls, establishing a link between those miRNAs and the development of the disease.

*Oat* (Ornithine aminotransferase) converts arginine and ornithine into glutamate, an intracellular second messenger involved in the coupling of glucose metabolism and insulin secretion. The *Oat* 3′ UTR is fairly short (∼750 nt), but contains predicted binding sites for several miRNAs, including miR-200b/c, a miRNA family that has been involved in the development of T2D (Belgardt et al., 2015). Nevertheless, to the best of our knowledge, experimental studies to identify miRNAs targeting *Oat* have not been performed so far.

A more detailed discussion of the function of these and other disallowed genes and their impact on β-cell identity is provided by Pullen and Rutter elsewhere in this issue.

## MiRNAs and β-Cell Responses to Stress

A miRNA typically targets several genes simultaneously whereas a given gene is generally targeted by various miRNAs at the same time. The main “duty” of miRNAs is thus to confer robustness and flexibility to cellular systems, buffering gene expression and enabling cells to maintain and transmit their states to daughter cells (Ebert and Sharp, 2012; Siciliano et al., 2013). It is not, therefore, surprising that loss of miRNA function *in vivo* rarely results in strong phenotypes under normal conditions, but that these become evident under stress situations. MiRNAs and stress signals can form intricate networks which might diminish or potentiate the stress signals, or even affect the threshold at which these are activated (Ebert and Sharp, 2012; Siciliano et al., 2013).

Throughout life, the β-cell is exposed to physiological stresses such as obesity (which may result in elevated levels of circulating fatty acids, pro-inflammatory cytokines, etc., as well as to hyperglycemia as β-cell function begins to be impaired), pregnancy, aging, growth, or genetic insulin resistance that might lead to endoplasmic reticulum (ER) stress, metabolic and oxidative stress, amyloid toxicity, inflammation or loss of cellular integrity. Failure of the β-cell to adequately respond to stress results in loss of β-cell function and mass (see above) and the extent to which loss of β-cell identity contributes to these effects is currently a matter of active debate (Butler and Dhawan, 2015; Rutter et al., 2015; Butler et al., 2016; Jeffery and Harries, 2016; Md Moin et al., 2016).

Given the central role of miRNAs in handling cellular stress and in stabilizing β-cell fate during development, it is anticipated that these small molecules may be involved in the response of the β-cell to stress. Researchers have therefore made a considerable effort to identify miRNAs whose expression is altered in β-cells by various types of stress and disease. Hence, variation in miRNA expression has been observed, for example, during compensatory β-cell expansion, during pregnancy and in obesity (Jacovetti et al., 2012; Tattikota et al., 2014), in islets from NOD mice, a model for cytokine-induced T1D (Roggli et al., 2012), islets from T2D murine models such as the Goto-kakizaki (GK) rat or *db/db* mice (Esguerra et al., 2011; Nesca et al., 2013), in human type 2 diabetic islets (Kameswaran et al., 2014; Locke et al., 2014), and during aging (Tugay et al., 2016).

Below, we discuss a potential role for miRNAs in maintaining β-cell identity and function during the most common stress situations that affect β-cells.

### Hyperglycemia, Hyperlipidaemia, and Diabetes

Central to the development of overt diabetes is the fact that high blood glucose negatively affects pancreatic β-cell function, producing a vicious circle that eventually results in complete β-cell failure (Jonas et al., 1999). The pathways underlying the deleterious effects of glucose on β-cells are far from being completely understood. Loss of the differentiated state of β-cells has been proposed as a major contributor to this failure (Jonas et al., 1999; Talchai et al., 2012; Jeffery and Harries, 2016). As such, glucose not only regulates insulin secretion, but also insulin mRNA transcription, stability and translation and it exerts a strong and long-lasting effect on gene expression (Schuit et al., 2002; Ren et al., 2007).

Early studies aimed to clarify the impact of glucose on the β-cell miRNome. Tang et al. (2009) used an array platform in a mouse β-cell line (MIN6) and found that the expression of dozens of miRNAs changed when these cells were cultured at various glucose concentrations for as little as 16 h. Of the miRNAs affected, only miR-124a, miR-107, miR-30a,d (up-regulated), and miR-690 (down-regulated) were further validated by RT-qPCR (Tang et al., 2009). MiR-30d stimulates insulin mRNA expression and is down-regulated in *db/db* mouse islets (Zhao et al., 2012). Suggesting a role as a positive regulator of β-cell identity, miR-30d indirectly promoted *MafA* expression, although it did not affect the expression of other transcriptional regulators such as *Pdx1* and *NeuroD* (Tang et al., 2009; Zhao et al., 2012). Although, it is believed that the effect of miR-30d on *MafA* is at least partially mediated by its direct target MAP4K4 (Zhao et al., 2012), other miR-30d targets in β-cells are as yet unidentified.

Studies focused in specific miRNAs have identified several other miRNAs that are regulated by glucose. For example, miR-375 is down-regulated in a rat insulinoma cell line (INS1) cultured at high glucose concentration during 48 h (El Ouaamari et al., 2008). Surprisingly, miR-375 expression is rapidly down-regulated in rat islets exposed to high glucose but strongly up-regulated following a longer period of incubation (El Ouaamari et al., 2008). MiR-375 expression is also reduced in diabetic GK rat islets (El Ouaamari et al., 2008). A similar pattern has been observed for miR-15a, a miRNA that promotes insulin biosynthesis by targeting uncoupling protein-2 (UCP-2) (Sun et al., 2011). MiR-15a is up-regulated in mouse islets exposed to high glucose for 1 h but down-regulated by a longer incubation with the sugar. This plasticity may reflect the dual role of glucose in β-cell function: glucose is required for adequate insulin secretion whereas chronic exposure of β-cells to high glucose concentrations eventually results in β-cell failure (glucotoxicity).

Additional examples are miR-184, whose role in the β-cell will be discussed below, negatively regulated by high glucose in mouse and *Drosophila* (Tattikota et al., 2015) and miR-133a and miR-146, up- and down-regulated by glucose in human islets, respectively (Fred et al., 2010). The latter miRNA, as well as miR-34a, are also modulated by other stressors such as cytokines (Lovis et al., 2008b; Fred et al., 2010) and are up-regulated in diabetic *db/db* mouse islets (Lovis et al., 2008b). Whereas miR-133a induction mediated the hyperglycemia-induced repression of insulin biosynthesis (Fred et al., 2010) and could therefore be involved in β-cell differentiation, miR-34a and miR-146 are probably implicated in β-cell survival (Lovis et al., 2008b). Further supporting a role for miRNAs in mediating the effects of nutrients on β-cell differentiation and function, Regazzi et al. have recently demonstrated that the expression of several miRNAs such as miR-29b, miR-17 and miR-25, changes during the post-natal β-cell maturation that occurs at weaning (Jacovetti et al., 2015). Nutrient changes associated with weaning have been demonstrated to trigger complete β-cell maturation, at least in mice, required for adequate β-cell secretion and compensatory proliferation (Stolovich-Rain et al., 2015).

Considerable efforts have been directed toward the identification of miRNAs altered in diabetic human islets. Using RT-qPCR-based arrays, Locke et al. (2014) detected an increase in the expression of miR-187 and miR-345 in T2D islets versus control. In this study we demonstrated that miR-187 can control glucose stimulated insulin secretion in INS1 cells, although an effect in beta-cell identity remains to be evaluated.

Two years ago, Kaestner’s group compared the expression of miRNAs between islets from diabetic and healthy donors using cutting-edge deep sequencing technology (Kameswaran et al., 2014). As well as confirming the large changes in miR-187 demonstrated by Locke et al. (2014), this approach allowed the authors to identify a miRNA cluster (DLK1-MEG3) dramatically down-regulated in diabetic islets. DLK1-MEG3, one of the largest human miRNA clusters, is a maternally imprinted locus at human chromosome 14q32 that contains several coding-genes, snoRNAs and 54 miRNAs (Benetatos et al., 2012). Several of those miRNAs (13) were down-regulated in the T2D islets (Kameswaran et al., 2014). The miRNAs from the DLK1-MEG3 cluster were on average expressed at much higher levels in sorted beta- than in alpha-cells, probably due to a loss of the repressive H3K27me3 marks in the promoter of this locus (Kameswaran et al., 2014). Moreover, islets from T2D donors contained increased DNA methylation in a differentially methylated region (DMR) overlapping with the MEG3 promoter which is responsible of the maternal imprinting (Kameswaran et al., 2014). Using HITS-CLIP (High-throughput Sequencing following Crosslinking and Immunoprecipitation) with Argonaute 2 (Ago2), an essential component of miRISC, the same authors isolated the human islet miRNA targetome and identified over 12,000 mRNA targets for miRNAs as well as more than 450 miRNAs associated in miRISC. They also found that miRNAs target islet mRNAs through not only the 3′ UTR but also the CDS and, to a less extent, the 5′ UTR. Although, they didn’t experimentally identify all the mRNAs targeted by the miRNAs altered in T2D islets, they confirmed that IAPP (islet amyloid polypeptide), known to trigger β-cell death and dysfunction in T2D (Montane et al., 2012), is a target for miR-376a and miR-342.

Kameswaran et al. (2014) also made the important discovery that a small proportion of chimeric reads mapped simultaneously to a miRNA and an mRNA, establishing miRNA-mRNA pairs. The latter allowed them to identify other targets for miRNAs of the DKL1-MEG3 locus. For example, they demonstrated that miR-495 targets the apoptosis-related gene *TPN53INP1*. *TPN53INP1* is the nearest gene to a SNPs associated with T2D (Voight et al., 2010), and global inactivation in mice leads to glucose intolerance reflecting impaired insulin sensitivity which is uncompensated by changes in β-cell function or mass (Seillier et al., 2015). Gene ontology analysis of the mRNAs identified in miRISC showed enrichment in biological processes such as “protein localization and transport,” “protein ubiquitination,” “regulation of cell death,” and “phosphorous metabolic processes.” It would be of great interest to interrogate these data for genes characteristic of β-cell differentiation and de-differentiation, including transcription factors, neuronal genes, or those characteristic of other endocrine cells or precursors.

### Pregnancy, Obesity, and β-Cell Compensation

During obesity and pregnancy, a decrease in insulin sensitivity raises the body’s need for insulin. In both cases, β-cells need to compensate for a higher insulin requirement that otherwise would lead to gestational and/or T2D. Compensation is characterized by an increase in the number and the secretory activity of the β-cells (Singh, 2016).

Work by Regazzi and co-workers group has greatly contributed to our understanding of the role of miRNAs during these compensatory processes. Using microarrays and RT-qPCR this group identified several rat islet miRNAs whose expression was altered during gestation (Jacovetti et al., 2012). Of those, miR-218, miR-338-3p, and miR-874 were up-regulated, whereas the expression of miR-144 and miR-451 was reduced. Of note, the targets of these miRNAs are diverse: miR-338 affected mouse islet cell proliferation (although its effects were not recapitulated in humans) whereas miR-338 and miR-451 were involved in the detriment effects of cytokines and palmitate on the cells (Jacovetti et al., 2012). Interestingly, Talchai reported that multiparous mice with FoxO1 ablation displayed increased number of β-cells that have loss insulin expression, and thus their identity (Talchai et al., 2012). A proportion of these cells retained expression of transactivators of insulin gene expression (e.g., *Pdx1* and *MafA*), but concomitantly expressed glucagon, somatostatin, or pancreatic polypeptide. Further studies are necessary to fully understand whether the above miRNAs are required for normal compensatory responses or whether changes in their expression occur as a consequence of metabolic stress.

It is also a strong possibility that the miRNAs involved in β-cell compensatory response differ depending on the type of stress that stimulates compensation. For example, early studies by Poy et al. (2009) demonstrated that miR-375 is required for β-cell compensation in leptin-deficient mice, whereas the study by Jacovetti et al. (2012) did not find differences in the expression of this miRNA during pregnancy. Another example is miR-184, whose expression is strongly decreased in mouse models of obesity and insulin resistance as well as in human T2D islets (Tattikota et al., 2014) but not during pregnancy (Jacovetti et al., 2012). The relevance of miR-184 silencing during β-cell compensation has mainly been attributed to one of its targets, *Ago2*, an essential mediator of miRNA function and the β-cell compensatory proliferation (Tattikota et al., 2014). Another important target of miR-184 is the glutamate transporter *Slc25a22*, which plays a major role in the effect of miR-184 in insulin secretion and mitochondrial function (Morita et al., 2013; Tattikota et al., 2015). Even though miR-184 overexpression in MIN6 cells did not exert a strong impact on gene expression, a certain degree of de-differentiation was hinted at by the up-regulation of *Ngn3* and *Ppy* and the down-regulation of *MafA* (Tattikota et al., 2015). Additional studies are required to determine if this de-differentiation occurs upon stress conditions *in vivo* and its impact in β-cell function.

### Aging

T2D is an age-related disease, and aging represents a major risk factor (De Tata, 2014). Age appears to contribute to increased insulin resistance, impaired β-cell insulin secretion (but see below), β-cell senescence and reduced β-cell proliferation (De Tata, 2014). Nevertheless, the extent and causes of these defects remain contested. Moreover, many of the mechanisms that contribute to the impairment of β-cell function and survival with age have been established only in murine models. However, it has recently become apparent that these might not be extrapolated directly to humans. For example, whereas in both rodents and humans β-cell proliferative capacity decreases with age (Teta et al., 2005; Maedler et al., 2006; Parnaud et al., 2008), proliferation of β-cells is almost undetectable after adulthood in man (Perl et al., 2010), at least outside of pregnancy (Butler et al., 2010). The impact of age in the secretory capacity of the β-cell is also unclear in both rodents and humans (De Tata, 2014). Nonetheless, the expression of genes essential for maintenance of the β-cell differentiated status, such as *FOXO1* and *PDX1* decreases with age in both murine and human islets, respectively (Kitamura et al., 2002; Maedler et al., 2006). This suggests that some loss of β-cell identity occurs with age. In fact, as with multiple pregnancy, aging mice with β-cell-specific *Foxo1* inactivation display a significantly higher number of β-cells that have loss insulin expression and adopted an α, δ- or PP-cell identity (Talchai et al., 2012).

To shed light into the role of miRNAs during aging, Tugay et al. (2016) used microarrays to profile miRNAs expressed in 3- and 12-month rat islets. This study allowed the authors to demonstrate that the expression of as many as 69 miRNAs was altered with age. Of these, miR-124a, miR-383, miR-34a (up-regulated) and miR-181a and miR-383 (down-regulated) were subsequently confirmed by RT-qPCR. MiR-34a had previously been associated with aging in other tissues and organisms (Li et al., 2011; Liu et al., 2012) and demonstrated to control insulin secretion and islet cell survival (Roggli et al., 2010). Suggesting a conserved regulatory mechanism in humans, miR-34a expression also correlated with age of human islet donors (Tugay et al., 2016). Interestingly, modulation of miR-34a, miR-383, and miR-130b strongly impact the response to different age-related apoptotic stimuli (Gunasekaran and Gannon, 2011; Tugay et al., 2016). Intriguingly, Tugay et al. (2016) found that 12-month old islets, although fully functional, failed to proliferate in response to mitotic stimuli such as exendin-4, PDGF or prolactin and demonstrated that miR-181a down-regulation or miR-34a up-regulation inhibited exendin-4 or PDGF-AA -stimulated proliferation. Pathway analysis of protein-coding genes concurrently affected by age identified, between many others, enrichment in pathways involved in the establishment, maintenance or loss of β-cell identity including MAPK, HIF-1 and FoxO signaling, as well as several neuronal-related pathways (Tugay et al., 2016).

Although, a link between age-dependent miRNAs and these pathways remains to be investigated, this study did allow us to demonstrate that miR-34a targets the disallowed gene *PDGFRA*, whose expression is reduced in aged rat islets, limiting their proliferation (Chen et al., 2011; Tugay et al., 2016).

## Perspectives for Future Study

### Mechanisms Underlying Regulation of miRNA Expression in β-Cells

Similar to protein-coding genes, miRNAs are susceptible to epigenetic and transcriptional regulation. Although the field of β-cell epigenetics is still in its infancy, examples in which miRNA expression is epigenetically controlled during development and disease have already started to emerge: as mentioned above, miRNAs from the DLK1-MEG3 cluster are regulated by differential DNA methylation around the MEG3 promoter in T2D islets (Kameswaran et al., 2014). Similarly, others (Hall et al., 2014) found that lower methylation of two miRNAs in the chromosome X, miR-660 and miR-532 correlated with higher expression levels in female versus male pancreatic islets.

In general, expression of pri-miRNAs is controlled by the same type of promoters as protein-coding genes, and involves the presence of distal enhancers. Considerable experimental and computational efforts have been directed toward the identification of pri-miRNA transcription start sites (TTS) (Marson et al., 2008; Chien et al., 2011; Nieto et al., 2012; Georgakilas et al., 2014). Nevertheless, TSSs can be located up to 100s of kilobases away from the corresponding mature miRNA which adds to the fact that pri-miRNAs are short-lived due to their rapid processing into mature miRNAs (Ha and Kim, 2014). Thus, TSSs haven’t been accurately identified for most miRNAs, neither have been the sequences responsible for their transcriptional regulation. Nonetheless, specific examples demonstrate that transcription factors, as well as being target of miRNAs themselves, control miRNA expression in islets. Thus, binding of *Pdx-1* and *NeuroD1* to the miR-375 *locus* has been suggested to play a critical role during β-cell maturation (Keller et al., 2007).

Micro RNA expression is subjected to additional levels of control throughout the multi-stepped processing of their primary transcripts. As recently reviewed by Connerty et al. (2015), several RNA-binding proteins (RBPs) drive miRNA maturation from their precursors. Thus, *DROSHA* and *DGCR8* are responsible for pri-miRNA cleavage into the intermediate ∼80 nt pre-miRNA in the nucleus, which is further processed by *DICER* and other co-factors such as *PACT* and *TRBP* in the cytosol. The important members of miRISC, argonaute proteins, not only are essential for miRNA-mediated target repression but have also been demonstrated to affect miRNA maturation in mammals (Diederichs and Haber, 2007; Czech and Hannon, 2011). In human islets, DICER is controlled by glucose (Schrimpe-Rutledge et al., 2012), whereas AGO2 has been proposed as an important player in β-cell compensatory expansion (Tattikota et al., 2014). Although, to the best of our knowledge, the regulatory mechanisms controlling miRNA-processing in the β-cell have not so far been investigated, several miRNAs with a role in β-cell identity are regulated at the pri/pre-miRNA processing level in other cell types. Examples are the already mentioned miR-7 (see above), miR-34a (Doridot et al., 2014; Herbert et al., 2014) or miR-146a (Srivastava et al., 2015).

Likewise, miRNA stability can be modulated in response to specific cellular stimuli or in a context-specific manner. MiRNAs are in general very stable, with an average half-life of ∼120 h (Gantier et al., 2011) but they can be subject to post-transcriptional modifications such as 3′-adenylation, 3′-uridylation and 2′-*O*-methylation, that may impact miRNA stability (Towler et al., 2015). Moreover, RBPS can also alter miRNA half-life (Diederichs and Haber, 2007; Towler et al., 2015), which can be further affected by other non-coding RNAs acting as miRNA sponges (Thomson and Dinger, 2016). As mentioned above, miR-7 represents an example of this type of regulation in β-cells. MiR-7 can be sequestered by a circular RNA acting as a miR-7 sponge (Xu et al., 2015). Whether and how miRNAs contribute to the roles in controlling β-cell identity of nutrient-sensing systems such as AMP-activated protein kinase (AMPK)- (Sun et al., 2010; Kone et al., 2014) and Per-Arnt-Sim (PASK)- (da Silva Xavier et al., 2004; Semplici et al., 2016) is also an intriguing area for future research.

Last but not least, RBPs can also modulate the efficacy of miRNA repression by affecting the structure of the target mRNA or the efficiency of miRISC recruitment (Kedde et al., 2007; Gulyaeva and Kushlinskiy, 2016). Accordingly, differential miRNA-mediated regulation in response to stress or metabolic signals can potentially occur without observable changes in miRNA expression.

Whereas, there is little doubt that epigenetic and transcriptional regulation of miRNA expression plays a central role in the establishment, maintenance and loss of cellular identity, studies on the mechanisms underlying miRNA processing, post-transcriptional modifications, stability and efficacy have only started to emerge. Future research tackling these questions will provide essential insights into the mechanisms governing miRNA function in the β-cell.

### MiRNAs and Generation of Functional β-Cells for Replacement Therapy in Diabetes

In recent years, several strategies have been envisaged to improve pancreatic islet function and identity in both Type 1 diabetes (T1D) and T2D. These were mainly based on (1) replacement of individual β-cells through human islet transplantation (2) stimulation of existing β -cell proliferation (3) prevention of β -cell death (i.e., blockade of apoptosis) (4) large-scale production of β-cells from multipotent progenitors and (5) expansion human of islet cells. Due to the low efficacy of islet transplantation mostly attributed to the rejection of transplants from hosts, some researchers turned their attention to the identification of pharmacological agents that stimulated β-cell proliferation. Whereas many groups identified ligands that increased β-cell mass in mice, very few of these showed beneficial effects in humans (Lovis et al., 2008a). One recently identified molecule that may have a promising future is SerpinB1, a liver-derived secretory protein, which enhances β-cell proliferation in zebrafish and mice and also stimulates the proliferation of human islets (Lovis et al., 2008b). Furthermore, TGFβ inhibitors were recently found to reverse the dedifferentiation process of expanded mouse (Blum et al., 2014) and human β-cells (Toren-Haritan and Efrat, 2015), revealing a degree of plasticity of dedifferentiated β-cells which could potentially be targeted by drugs to improve β-cell identity.

A considerable body of research into the intricate mechanisms governing β-cell differentiation during development, and recent advancements on somatic transfer and cell reprograming, have paved the way to the production of β-cells from hESC and induced pluripotent stem (iPSC) cells (Plaisance et al., 2006; Liao et al., 2013; Park et al., 2013). Whereas production of β-cells *en masse* following the differentiation of hESC and iPSCs may allow a more personalized therapy for diabetes, many challenges are in front of us before stem cell-based diabetes therapies can be widely used by patients with diabetes (reviewed in Quiskamp et al., 2015). Whether, such strategies could be used in combination of drugs that normalize the identity of existing β-cells in diabetic patients still needs to be determined. Below we present recent advances describing how manipulation of miRNAs expression may act on β-cell identity and our ability to generate functional β-cells from multipotent progenitors and dissociated adult human islet cells.

### β-Cells from hESCs

In order to better understand the molecular components involved in the differentiation of pluripotent cells into insulin-producing cells, several groups have profiled the expression of miRNAs during differentiation of hESC using protocols established in recent years (D’Amour et al., 2005; Kroon et al., 2008; Nostro et al., 2011). These studies revealed that two of the most highly expressed miRNAs in adult liver and pancreas, namely miR-122 and miR-375, are also amongst the most highly induced miRNAs during the differentiation of ESCs into definitive endoderm (Tzur et al., 2008; Hinton et al., 2010; Kim et al., 2011; Francis et al., 2015). MiR-30d and miR-200a are also induced during the differentiation of hESC into definitive endoderm whereas miR-151a-5p and miR-151-a-3p were up-regulated in differentiated hESCs (Migliorini et al., 2014; Francis et al., 2015). Chen et al. group have shown that miR-186, -199a, and -339 are found at higher levels in islet-like insulin-positive cell clusters derived from the differentiation of hESC (Joglekar et al., 2009b). Interestingly, acquisition of insulin expression in differentiated hESCs correlates with increased expression of miR-375 (Liao et al., 2013; Wei et al., 2013) and miR-375 overexpression is sufficient to promote pancreatic endocrine differentiation from hESCs in the absence of any extrinsic inducers (Jopling et al., 2005). In fact, overexpression of miR-375 of mesenchymal stem cells obtained from either human bone marrow or human placenta can also redirect them into functional insulin-expressing cells (Bazzini et al., 2012; Shaer et al., 2014b). These studies illustrate the crucial role of miR-375 in governing for the initiation phase of ESC differentiation and acquisition of insulin expression in β-cell precursors.

### β-Cells from iPSCs

Induced pluripotent stem cells generated from the reprogramming of skin fibroblasts (Maekawa et al., 2011) have also been widely used for the generation of endoderm and insulin-producing cells. Porciuncula et al. (2013) have measured the expression of miRNAs during induction of iPSCs into endoderm and identified 13 up-regulated miRNAs including miR-18a -103, -206, -302a/c and the islet-enriched miR-141 and miR-200c. Conversely, reprogramming of skin fibroblasts isolated from T1D patients in iPSCs is accompanied with the induction of pancreas-enriched miR-7, miR-9, and miR-375 compared to parental fibroblasts, strengthening the role of these miRNAs in human pancreatic progenitors (Liu et al., 2014). Together, these studies suggest that modulation of miRNAs levels may affect the ability of multipotent stem cells to differentiate into insulin-positive cells. Conversely, overexpression of miR-186 and miR-375 by chemical transfection of human iPSCs promoted islet-like cell cluster formation associated with the induction of markers found in endocrine progenitors (Ngn3) and mature β-cells (Insulin, Pdx1, Pax4/6, Nkx6-1, Glut2, and Kir6.2) (Shaer et al., 2014a). Interestingly, the same group obtained similar results when miR-7 was overexpressed (Shaer et al., 2016). Virus-mediated overexpression of miR-375 in human skin fibroblast-derived iPSCs is sufficient to trigger their differentiation into insulin-expressing cells and allow glucose-dependent insulin secretion *in vitro* (Lahmy et al., 2014). Finally, hESCs overexpressing miR-410, miR-495, and miR-590 that have subsequently been subjected to differentiation into endoderm can improved glycemic control when transplanted in mouse models of gestational or T2D diabetes (Chen et al., 2015; Liang et al., 2015; Mi et al., 2015). Interestingly, Ldha and Mct1/Slc16a1, two disallowed genes in β-cells, are repressed by these miRNAs in hESCs (Chen et al., 2015; Liang et al., 2015; Mi et al., 2015), demonstrating that miRNA-mediated regulation of disallowed genes is also of functional importance during the differentiation of pluripotent cells into β-cells. Together these studies reveal the great therapeutic potential of miRNAs in stem-cell-based approaches for treatment of diabetes.

### β-Cells from Expansion of Human Islets

One approach to counteract the shortage of islet donation relies on the expansion of dissociated human β-cells which is, however, limited by the low proliferation rate of these cells and by the loss of β-cell phenotype (i.e., de-differentiation) that occurs during their *in vitro* propagation reviewed in (Efrat, 2008). Growth signals were found to trigger an epithelial-mesenchymal transition (EMT) process in those cells which correlated with the activation of TGFβ (Toren-Haritan and Efrat, 2015), Notch (Bar et al., 2008) and Wnt (Lenz et al., 2014) signaling pathways. Nathan et al. (2015) analyzed changes in miRNA expression following expansion of human adult β-cells undergoing such de-differentiation process and found that expression of miR-375, miR-192, miR-204, miR-215 and the miR-200 and miR-30 families is down-regulated compared to undifferentiated control β-cells. Importantly, overexpression of miR-375 induced the expression of several key transcription factors such as *PDX1*, *MAFA*, *NKX6.1* and *PAX4*, indicative of re-differentiation. Repression of the PDK1-AKT pathway by miR-375 mediated this re-differentiation process at least to some extent (Nathan et al., 2015). Whether restoring the expression of other miRNAs altered in de-differentiated β-cells promotes their re-differentiation remains to be evaluated, but the above studies suggest that modulation of miRNA expression in dissociated human β-cells may prevent their dedifferentiation in culture. As such, this strategy may provide a new therapeutic avenue for the generation of functional insulin-producing cells.

### MiRNAs and β-Cell Heterogeneity

Although, several lines of evidence support a role for miRNAs in regulating the identity of pancreatic β-cells, these observations need to be interpreted with caution especially in light of recent studies demonstrating a high degree of heterogeneity between individual β-cells. The functional diversity of individual β-cells has been suspected for many years (Salomon and Meda, 1986; Van Schravendijk et al., 1990), but the functional significance of these differences, or the possibility that certain cells may play a pacemaker role, has been difficult to assess. Johnston et al. (2016) used a combination of high speed Ca^2+^ imaging (Hodson et al., 2013) and optogenetic inactivation to demonstrate the existence of “hubs” or “pacemaker” β-cells that impose synchronicity on other “follower” cells in mouse islets. These cellular hubs display a distinct expression profile to follower cells that is partly shared with immature β-cells and associated with low insulin, *Nkx6-1* and *Pdx1* expression, but high *Gck* levels.

Whilst the study by Johnston et al. (2016) did not analyze the transcriptional profile of “hub” or “follower” cells in depth, others have recently showed that subpopulations of β-cells exist within human islets and display a distinct genetic signature with a specific subtype over-represented in diabetes (Bader et al., 2016; Dorrell et al., 2016). Indeed, single cell RNA sequencing (RNA-Seq) has extended these findings to demonstrate heterogeneity at the level of individual human β-cell transcriptomes (Segerstolpe et al., 2016). How and when such heterogeneity is created during development still remains to be fully elucidated, and the role of miRNAs unknown. Of note, heterogeneity was observed several years ago in primary and distal tumors found at secondary sites as well as within cells of a tumor (Heppner, 1984). Interestingly, an intra-colorectal tumor gradient has been found for miR-375 and miR-200c, two miRNAs enriched in pancreatic β-cells (Jepsen et al., 2016). Given that several miRNAs modulate the identity of β-cells through repression of a plethora of pancreatic transcriptional regulators and enzymes required for the function of the mature cell (see above), it is possible that the differential expression of miRNAs plays a role in establishing cellular diversity within islets. It remains, however, to be investigated whether discrete subpopulations of pancreatic β-cells display distinct miRNA gene expression profiles. Nonetheless, with the advance of single cell RNA-Seq (Segerstolpe et al., 2016; Wang Y.J. et al., 2016; Xin et al., 2016), we are likely soon to have answers to these questions.

## Author Contributions

AM-S, GR, and ML drafted, wrote and critically revised the manuscript.

## Conflict of Interest Statement

The authors declare that the research was conducted in the absence of any commercial or financial relationships that could be construed as a potential conflict of interest.

## Footnotes

1 www.targetscan.org
